# Predictors of Women’s awareness of common non-communicable diseases screening during preconception period in Manna District, Southwest Ethiopia: implication for wellness check-up

**DOI:** 10.1186/s12913-021-06067-2

**Published:** 2021-01-12

**Authors:** Firanbon Teshome Gonfa, Yohannes Kebede Lemu, Zewdie Birhanu Koricha

**Affiliations:** Department of Health, Behavior, and Society, Faculty of Public Health, Jimma University, Jimma, Ethiopia

**Keywords:** Non-communicable diseases, Awareness, Preconception care, Ethiopia

## Abstract

**Background:**

Non-communicable diseases (NCDs) are the dominant cause of global morbidity and mortality, especially in developing countries. Tackling NCDs is central to advancing women’s and child health, and future generations. Many NCDs can be prevented with appropriate approaches across the maternal and child health life-cycle, throughout the years of reproductive age especially before conception and continuing through pregnancy. However, women’s awareness of NCDs screening during the preconception period was not well known in many countries including Ethiopia. Therefore, this study aimed to assess women’s awareness of common NCDs screening during the preconception period and associated factors in Manna District, Jimma Zone, Oromia Region, Ethiopia, 2019.

**Methods:**

A community-based cross-sectional study was conducted from March 02 to April 10, 2019. The sample size was 636 pregnant women from eight randomly selected rural kebeles and a purposively taken urban kebele. The data were collected using a pre-tested structured questionnaire and entered into Epi data manager version 4.0.2 and exported to SPSS version 21. Descriptive, binary, and multivariable logistic regression analyses were carried out.

**Results:**

Of the total of 623 respondents, 459 (73.7%) of them had good awareness of common NCDs screening during the preconception period. Women who had formal education [AOR = 1.95, 95% CI: (1.31–2.89)], those who had planned pregnancy [AOR = 2.17, 95% CI: (1.47–3.19)], on ANC follow up [AOR = 1.79, 95% CI: (1.16–2.74], and those who had media (radio and/or television) in their house [AOR = 1.55, 95% CI: (1.05–2.29)] had good awareness on common NCDs screening during the preconception period compared to their counterparts.

**Conclusions:**

In this study, nearly three-quarters of respondents had a good awareness of common NCDs screening during the preconception period. Women’s educational status, pregnancy planning status, ANC visit, and having radio and/or television in the house were predictors of women’s good awareness of common NCDs screening during the preconception period. Therefore, all concerned bodies are recommended to work toward increasing women’s awareness using different approaches like awareness creation campaigns and counseling clients attending health facilities.

## Background

Non-communicable diseases (NCDs) are the leading public health challenges globally and cause more than 71% of worldwide deaths [1]. More than three-fourths of all NCD deaths and 85% of premature NCD deaths occur in developing countries [2], which can be due to people from low-income countries are more exposed to risk factors and have less access to health services [3]. The four common NCDs which accounts for 80% of NCD deaths are cardiovascular diseases, cancers, chronic respiratory diseases, and diabetes [1]. These NCDs are due to the four main modifiable risk factors. Namely: tobacco use, unhealthy diets, physical inactivity, and harmful use of alcohol [4, 5]. If these risk factors are eradicated, more than three fourth of the four common NCDs can be prevented [6].

NCDs are expected to exceed infectious diseases as major causes of morbidity and mortality in Africa by the year 2035 [7]. Evidence indicated that in Africa, 30.8% and (6.3–15.4%) of people had hypertension and diabetes mellitus, respectively [8, 9]. Anemia among all women of reproductive age accounts for 38.6% [10]. Similarly, Ethiopia is also suffering from NCDs. Evidence from Ethiopia showed that 95% of the study population was found with 1–2 NCD risk factors [11], 19.6% of the population had hypertension [12], 3.8% had diabetes [13], and 23% of women of reproductive age had anemia [14].

NCDs affect people of all age groups [15], and put burdens on individuals, families, and communities, which had social, economic and public health impacts, and impair quality of life [16, 17]. Despite, both males and females affected, evidence indicated that NCDs had a great impact on women’s lives than men [18, 19]. NCDs are the leading cause of death for women worldwide (account for almost 65% of women’s deaths), and over three fourth of these deaths occur in low- and middle-income countries (LMICs) [20]. Each year, 35 million deaths result from NCDs and among those, 18 million deaths occur in women, often in their most productive years [21, 22]. It also impedes progress toward sustainable development goals (SDG), mainly SDG 1, 3, 4 and 5 [23]. NCDs among women of reproductive age has doubled in many African countries [24]. Majority of them are due to modifiable risk factors [4, 5]. For instance, in addition to the immediate complications on pregnancy, maternal obesity can results in coronary heart disease, stroke, type 2 diabetes and asthma [25]. The risk of getting NCDs highly increases during pregnancy, which leads to serious complications that threaten the health and lives of mothers and their babies [23].

The negative consequences of NCDs include: psychological burden, poor self-efficacy, end-organ damage, pre-eclampsia, miscarriage, hemorrhage, cesarean sections, post-natal complications, intrauterine growth restriction, preterm labor, birth injury, neural tube defects, stillbirth, low birth weight, neonatal hypoglycemia, infant respiratory distress syndrome, fetal death, and perinatal death [26–28]. They can also result in long term effects like increased risks of infertility, long term disability, impaired life chances, poor educational performance, loss of productivity and family income, increased risk of NCDs later in life, and premature mortality [23, 29]. Indeed, NCDs also cause stigma and discrimination [30]. Majority of all these can be prevented when preconception care is properly utilized.

Preconception care is the provision of biomedical, behavioral, and social health interventions to women and couples of childbearing ages before pregnancy occurs, which aimed at improving the health outcomes for women, newborns and children by reducing risk factors that could lead to poor maternal and child health outcomes [31]. It has paramount importance to reduce NCDs by providing an opportunity to early optimizing the health of potential mothers and couples [31, 32]. The World Health Organization (WHO), Centers for Disease Control and Prevention (CDC), and International Federation of Gynecology and Obstetrics (FIGO) recommends the need for women to screen for NCDs before conception which helps for early identification of the diseases, management, and reducing its consequences [31, 33, 34]. There are also evidences of the effectiveness of preconception care. For instance, a systematic review and meta-analysis from Pakistan indicated that maternal diabetic care during preconception period reduces the occurrence of congenital malformations by 70% and perinatal mortality by 69% [35]. However, despite its importance and recommendations [31, 33, 34], to the best of the authors’ knowledge, there are no published articles on awareness of NCDs screening during the preconception period in Ethiopia. Therefore, this study aimed to assess women’s awareness of common NCDs screening during the preconception period and associated factors in Manna district, Jimma Zone, Oromia region, Ethiopia, 2019.

## Methods

### Study design, area, and period

A community-based cross-sectional study was conducted from March 02/2019 to April 10/2019 in the Manna district among pregnant women. The Manna district is one of the 21 districts found in Jimma zone, Oromia Region. It is located 368 km southwest far from Addis Ababa (the capital city of Ethiopia) and 22 km from Jimma town. According to the 2019 report obtained from the Manna District Health Office, the district has a total population of 197,911, of which 26,451 were urban and 171,460 were rural. Women of reproductive age groups of the district were 43,738, and pregnant women were 6868. The district has a total of 26 kebeles: 1 urban and 25 rural kebeles. It has 7 health centers, 26 health posts, 11 private clinics, and 3 private pharmacies. It has also 68 health extension workers and 121 health care providers of different professions.

### Population

The source populations were all pregnant women found in the district during the study period, and the study populations were randomly selected pregnant women who fulfilled the inclusion criteria. All pregnant women (regardless of the gestational age) who lived in the district for at least 6 months prior to the study period were included in the study. Pregnant women who were critically ill and unable to communicate were excluded. In this study, pregnant women were recruited instead of women in preconception period due to issues related to feasibility, as list of pregnant women were easily obtained from the family folder of the community health information system.

### Sample size and sampling procedures

The sample size was determined by using a single population proportion formula, considering the following assumptions: 50% proportion of women’s awareness of NCDs screening during the preconception period since there was no prior study in Ethiopia specifically to address the study objectives, 95% level of confidence, 5% margin of tolerable sampling error, 10% non-response, and 1.5 design effects. Based on these, the final sample size of the study was 636. To select the study participants, first, the 26 kebeles were stratified into rural and urban. Then, the urban kebele was included in the study purposively for representation. Eight kebeles among the 25 rural kebeles were selected using a simple random sampling technique. Then, the sample size was proportionally allocated to the selected 9 kebeles. Accordingly, a total of 566 pregnant women (Haro = 89, Gudeta Bula = 42, Buxure = 54, Somodo = 83, Gube Muleta = 65, Bilida = 86, Kenteri = 72, and Sombo Manna = 75) were allocated to the eight selected rural kebeles. For the urban kebele (Yabbu town), 70 pregnant women were allocated. The lists of the total number of pregnant women found in the selected rural kebeles were obtained from the family folder of the community health information system, which is available at the health post. The rural health extension workers identify pregnant women, women on family planning and women who had under-five children and register them on the family folder. They update the family folder every month. For the urban kebele, since the family folder did not exist, a census was conducted by two urban health extension workers to construct the sampling frame. The two health extension workers conducted home to home visit and asked every woman of reproductive age whether they are on family planning or not, the absence of their menstrual period, its duration, early pregnancy symptoms and visits women’s abdomen to identify the pregnant women. The human chorionic gonadotropin (HCG) test was not done due to a lack of resources. Finally, computer-generated simple random sampling was used to identify the study participants. Their usual place of residence was identified in collaboration with kebele leaders.

### Data collection

Data were collected using an interviewer-administered structured questionnaire developed after reviewing different relevant literatures. It was first prepared in English and then translated to Afan Oromo and Amharic by experts. Then, it was translated back to English by another person to ensure its consistency and accuracy. A pretest was conducted among 5% of pregnant women in the Saka district, which is located 20 km away from the study area. A total of 6 data collectors (4 clinical nurses and 2 BSc nurses) and 2 public health officers as supervisors were recruited based on their previous experience in data collection and fluency in the languages of the community. In addition, the authors also closely supervised the data collection processes. The data collectors and supervisors were trained for 1 day on the objective of the study, data collection tool, approach to the interviewees, details of interviewing techniques, respect and maintaining privacy and confidentiality of the respondents. Cronbach’s α coefficient was computed to test the internal consistency of the tool, which was 0.88. The data collectors asked pregnant women about their awareness of NCDs screening specifically before they became pregnant.

### Study variables

Awareness of Common non-communicable diseases screening during the preconception period was the dependent variable. Socio-economic and demographic factors (age, residence, educational level, occupation, marital status, family size, and wealth of the household), gynecologic and obstetric factors (history of family planning use prior to conception, pregnancy planning status, parity, gravidity, and antenatal care visit), preexisting medical illnesses, health facility-related factor (distance from health facility), and media-related factors (access of radio and/or television in the household) were independent variables.

### Operational definitions

Kebele: The lowest government administrative hierarchy that exists next to district.

Common non-communicable diseases: In this study, the common non-communicable diseases are anemia, diabetes mellitus and hypertension.

Good awareness of common non-communicable diseases screening during preconception period: defined as having ever “heard” or “read” about screening for at least one of the three common non-communicable diseases (anemia, diabetes mellitus and hypertension) for the sake of becoming pregnant. That means, in this study, women who answered “Yes” to at least one of the following three questions were considered as “women with good awareness of common non-communicable diseases screening during preconception period”. Questions:

1. Have you ever heard or read about screening for hypertension for the sake of becoming pregnant?
2. Have you ever heard or read about screening for diabetic mellitus for the sake of becoming pregnant?
3. Have you ever heard or read about screening for low blood (anemia) for the sake of becoming pregnant?

### Data processing and analyses

After checking the completeness of the data manually, the collected data were entered, cleaned, and checked using Epi data manager version 4.0.2. Then, the data were exported to SPSS version 21 for analyses. Bivariable and multivariable logistic regression analyses were carried out to identify an association between the predictors and outcome variables. Binary logistic regression analysis was performed to select variables for multivariable logistic regression analysis. Variables with a *p*-value < 0.25 in the binary logistic regression analysis were taken as candidates for multivariable logistic regression analysis. Finally, multivariable logistic regression analysis was performed to control for the possible confounding effects of the selected variables. Variables with a p-value < 0.05 were recognized as statistically significant associations with women’s good awareness of NCDs screening during the preconception period. Odds ratio with its 95% CI was used to show the degree of association between the outcome and independent variables. Descriptive analyses like frequencies and proportions were also conducted for different variables as necessary.

### Ethical consideration

A letter of ethical approval was received from the Institutional Review Board of Jimma University Institute of Health. The necessary permission was obtained from the Manna district health office and kebele administrative offices. All the study participants were informed about the purpose of the study, their right to refuse and assured about the confidentiality of the information they provided. Written informed consent was obtained from all the study participants. For participants under 18 years old, written consent was obtained from their parents.

## Results

### Socioeconomic and demographic characteristics of the study participants

A total of 623 pregnant women participated, giving a response rate of 98.0%. More than half of the respondents, 352(56.5%) were in the age range of 25–34 years. A majority of the respondents, 553(88.8%), 583(93.6%), and 462(74.2%) lived in rural areas, Muslims by religion, and housewives in their main occupation, respectively. More than half, 328(52.6%) of the respondents had no formal education, whereas only a few, 8(1.3%) attended college- or university-level education (Table 1).

**Table 1:** Socio-demographic characteristics of respondents, in Manna district, Jimma zone, Oromia region, Southwest Ethiopia, 2019 (*N* = 623)

Variable	Category	Frequency	Percent
Age of the respondents	15–24	196	31.5
	25–34	352	56.5
	35–49	75	12.0
Residence	Rural	553	88.8
	Urban	70	11.2
Religion	Muslim	583	93.6
	Orthodox	28	4.5
	Protestant	12	1.9
Ethnicity	Oromo	580	93.1
	Dawuro	21	3.4
	Amhara	14	2.2
	Other^a^	8	1.3
Educational level of the respondents	No formal education	328	52.6
	Primary education (1–8)	231	37.1
	Secondary education (9–12)	56	9.0
	Tertiary (college or university)	8	1.3
Main occupation of the respondents	Housewife	462	74.2
	Farmer	106	17.0
	Merchant	39	6.3
	Other^b^	16	2.6
Marital status	Married	618	99.2
	Other^c^	5	0.8
Wealth index	Lowest	215	34.5
	Middle	201	32.3
	Highest	207	33.2

^a^Kaffa, Gurage and Silxe ^b^Student, Daily worker, Private employee, and Government employee ^c^Single and separated

### Obstetric and gynecologic, and pre-existing medical illness of the study participants

Of the total of 623 respondents, 98(15.7%) of the women were pregnant for the first time. A majority, 421(67.6%) of the women were multiparous. More than half, 351(56.3%) of the women had a history of utilizing short-term family planning methods. One hundred thirty-two (21.2%) of the women had a history of four or more ANC visits, and 46(7.4%) had pre-existing medical illnesses. The current pregnancy was planned by 423(67.9%) of the women. (Table 2).

**Table 2:** Obstetric and Gynecologic factors of respondents in Manna district, Jimma zone, Oromia region, Southwest Ethiopia, 2019 (*N* = 623)

Variable	Category	Frequency	Percent
Gravidity	Primigravida	98	15.7
	Multigravida	525	84.3
Parity	Null parous	103	16.5
	Primiparous	99	15.9
	Multiparous	421	67.6
ANC visit	Not on ANC visit	217	34.8
	One visit	45	7.2
	Two visits	130	20.9
	Three visits	99	15.9
	Four or more visits	132	21.2
Previous history of Family planning use	Not on family planning	173	27.8
	Short term	351	56.3
	long-acting	99	15.9
Pregnancy planning status	Planned	423	67.9
	Unplanned	200	32.1
Preexisting medical illness	Yes	46	7.4
	No	577	92.6

### Women’s awareness of common NCDs screening during the preconception period

**Fig. 1:**
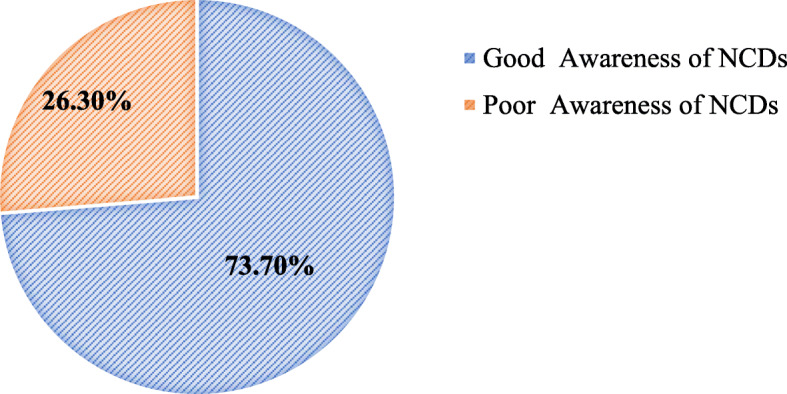
Women’s awareness of common NCDs screening during the preconception period in Manna District, Jimma Zone, Oromia Region, Southwest Ethiopia, 2019

Findings showed that, of 623 participants, 459(73.7%) reported that they had a good awareness of common NCDs screening during the preconception period *(*Fig. 1).

### Predictors of women’s awareness of common NCDs screening during the preconception period

All independent factors were checked for having an association with women’s awareness of common NCDs screening during the preconception period in bivariate logistic regression. Place of residency, women’s education, husband education, pregnancy planning status, history of family planning before pregnancy, ANC visits for current pregnancy, history of follow up for pre-existing illnesses, distance from the health facility, and media access (radio and/or television in their house) had shown as having an association with women’s good awareness on common NCDs screening during the preconception period. However, after controlling for confounding factors, only women’s education, pregnancy planning status, ANC visits for current pregnancy and media access in their house remained as predictor factors for women’s good awareness of common NCDs screening during the preconception period (Table 3).

**Table 3:** Predictors of women’s awareness of common NCDs screening during the preconception period in Manna district, Jimma zone, Oromia region, Southwest Ethiopia, 2019 (*N* = 623)

Variable	Awareness on NCDs		COR [95% C.I]	AOR [95% C. I]
	Good	Poor		
Residency				
Urban	61	9	2.64[1.28–5.45] *	1.50[0.68–3.31]
Rural	398	155	1.00	1.00
Women’s educational status				
Had formal education	242	53	2.34[1.61–3.40] **	1.95[1.31–2.89] **
No formal education	217	111	1.00	1.00
Husband educational status				
Had formal education	259	66	1.92[1.34–2.76] **	1.28[0.85–1.94]
No formal education	200	98	1.00	1.00
Pregnancy planning status				
Planned	339	84	2.69[1.86–3.90] **	2.17[1.47–3.19] ***
Not planned	120	80	1.00	1.00
F/P before current pregnancy				
Yes	338	111	1.35[0.91–1.98]	0.92[0.59–1.45]
No	120	53	1.00	1.00
Current status of ANC visit				
Yes	372	114	1.88[1.25–2.82] *	1.79[1.16–2.74] **
No	87	50	1.00	1.00
Hx of pre-existing illness				
Yes	39	7	2.08[0.91–4.75]	2.07[0.88–4.90]
No	420	157	1.00	1.00
Distance from health facility				
< 30 Minute	181	45	1.72[1.17–2.55] *	1.36[0.90–2.05]
≥30 Minute	278	119	1.00	1.00
Media				
Had media (TV/Radio)	211	53	1.78[1.22–2.59] *	1.55[1.05–2.29] *
No Media (TV/Radio)	248	111	1.00	1.00

*Statistically significant at *P*-value < 0.05, **Statistically significant at *P*-value < 0.01, ***Statistically significant at *P*-value < 0.001, *F/P* Family planning, *Hx* History

Findings of multivariable logistic regression analysis showed that the odds of having good awareness on common NCDs screening during the preconception period were nearly two times higher among women who had formal education compared to those who had no formal education [AOR = 1.95, 95% CI: (1.31–2.89)]. The odd of having good awareness of common NCDs screening during the preconception period were nearly two times higher among women who had planned pregnancy compared to those who hadn’t planned their pregnancy [AOR = 2.17, 95% CI: (1.47–3.19)]. Findings also indicated that ANC visit determined women’s awareness of NCDs screening. Women who were on ANC visits for their current pregnancy were 1.79 times [AOR = 1.79, 95% CI: (1.16–2.74] more likely to have good awareness on common NCDs screening during the preconception period compared to those who were not on ANC visit. The likelihood of having good awareness on common NCDs screening during the preconception period was nearly two times higher among women who had media (radio and/or television) in their house compared to their counterparts [AOR = 1.55, 95% CI: (1.05–2.29)] (Table 3).

## Discussion

NCDs are rapidly increasing and emerging as the leading cause of death, disability, reduced quality of life and rising health care costs worldwide [36, 37]. Screening for NCDs before conception has paramount importance in reducing the negative consequences of NCDs like adverse pregnancy outcomes and improving the health of women, children and future generations through early identification and management of diseases, risk factors [38]. The present study aimed to assess the magnitude of women’s awareness of common NCDs screening during the preconception period and associated factors. Accordingly, this study found that 73.7% of women had good awareness of common NCDs screening during the preconception period. Women who had a formal education, those who planned their pregnancy, being on ANC follow up and those who had media (radio and/or television in their house) had a significant association with women’s awareness on common NCDs screening during the preconception period.

The results of the current study revealed that 73.7% of the respondents had good awareness of common NCDs screening during the preconception period. This finding was comparable with the studies conducted in Nigeria (76.0%) [39] and Addis Ababa Ethiopia (72.5%) [40]. This might be due to the similarity of the study population. The previous and current studies were conducted among pregnant women. However, the finding of the current study was lower than studies done in Brazil (97.6%) [41] and Nepal (79.5%) [42]. The possible reason could be due to the difference in the health system and socio-cultural factors. In addition, the difference might also be due to the difference in study design and population. The study conducted in Brazil was facility-based. Women who attend the health facility for services like antenatal care, delivery, and postnatal care may have a greater chance of getting information about their health and preparation for the next conception. The study from Nepal was conducted among students of higher educational levels, whereas in the current study, more than half of the women had no formal education. As the level of education increases, the information exposure about health issues including preconception care also increases. Indeed, the difference might be due to lower media access in Ethiopia.

The current study was higher than studies conducted in upper income countries like US (57%) [38], Netherlands (65.2%) [43], Australia (40%) [44], and upper-middle-income countries like Brazil (67.9%) [45], Lebanon (53.5%) [46] and Jordan (50%) [47]. The observed discrepancy between studies might be due to the fact that the current study focused on a specific component of preconception care which is non-communicable diseases screening. However, previous studies assessed the overall awareness of preconception care. The difference might also be due to larger gaps in the study period between current and previous studies. The current study was also significantly higher than studies conducted in developing countries like Nigeria (63.5%) [48], Ghana (34.5%) [49], Kenya (25.3%) [50], Sudan (11%) [51], and West Shoa zone, Ethiopia (22.1%) [52]. This might be due to the fact that despite the early recommendation of WHO, CDC, and FIGO, preconception care is a new concept in many countries. Unequal attention was given even in different areas of a country. Another possible justification for this might be due to the larger sample size of the current study. Indeed, the difference might be due to gaps in the study period between current and previous studies.

Our study also identified predictors of women’s good awareness of common NCDs screening during the preconception period. In this study, women’s educational status determined their awareness of NCDs screening during the preconception period. Women who had formal education were nearly two times more likely to have good awareness of NCDs screening during the preconception period than their counterparts. This find was consistent with previous studies from different countries [40, 48, 49, 51–53]. Attending education gives individuals the chance of gaining information directly from the class or indirectly from discussion with friends or using different information sources like novel social media. Pregnancy planning status was another determinant factor of women’s awareness of NCDs screening before conception. The finding of the current study showed that women who planned their current pregnancy had good awareness of NCDs screening during the preconception period compared to those who had unplanned pregnancy. This finding was supported by studies from developing countries; Jordan and Wolayita Zone, South Ethiopia, which highlighted the positive effect of planned pregnancy status on women’s awareness of preconception care [47, 54]. This might be due to the fact that women who planned their pregnancy might have higher information-seeking behavior, contact health care providers and ask the prerequisites of conception. They might also gather information from media, friends and families.

The current study also identified that being on ANC follow-up helped women to have good awareness of NCDs screening during the preconception period. Women who were on ANC follow up were nearly two times more likely to have good awareness of NCDs screening during the preconception period than their counterparts. This finding was similar to studies done in Nigeria, Sudan, and Hawassa city, South Ethiopia [53, 55, 56]. This might be due to the reason that women on ANC follow-up might have a greater chance of getting information about preconception care, as health care providers may ask them about their health status before conception and provide counseling on the current and future pregnancies. Findings of the current study also showed that women who had radio and/or television in their house had good awareness of NCDs screening during the preconception period than those who had no radio and/or television in their house, which was consistent with studies conducted in Nepal and Wolayita Zone, South Ethiopia [42, 54]. This was due to the fact that media is the main source of information for different issues including health care services. News media (television, radio, print) and social media (including Twitter, Facebook, Instagram, YouTube, etc.) are top sources of reproductive health-related information and helps to improve maternal and child health [57, 58].

This study had several strengths. It is a community-based study that makes it a representation of the true population. Both urban and rural populations were included, which helped for the generalizability of the findings to the district as a whole. In addition, the findings of this study added evidence to the limited literature on awareness of preconception care among women. A study does not end without limitations. Recall bias may occur on some questions such as those related to obstetric and gynecologic factors as pregnant women were asked for history before they conceived. Interviewer bias may also have occurred. There might be pregnant women who didn’t register in the family folder. In addition, there might be the possibility of missing pregnant women during the census as the identification was oral-based. The human chorionic gonadotropin (HCG) test was not done due to lack of resources which was one of the potential limitation of this study. However, to mitigate this possibility, health extension workers were recruited to conduct the census. This helps to reduce the opportunities of missing pregnant women, as health extension workers know almost all women found in their kebele due to their repeated contact with women during home-to-home visits, conferences, and one to five networks. In addition, health extension workers have a friendly approach with women. Lack of literatures on awareness of non-communicable diseases screening during the preconception period was also another limitation for the study.

## Conclusion

In this study, nearly three-quarters of respondents had good awareness of common NCDs screening during the preconception period, indicating that still, more than one-fourth of the respondents had poor awareness. Women’s educational status, pregnancy planning status, ANC visit and media access (having radio and/or television in their house) were predictors of women’s awareness of common NCDs screening during the preconception period. These imply that there is a need to provide appropriate and adequate information on preconception care to potential mothers. Therefore, health extension workers, health care providers, local and national managers, non-governmental organizations, and media personnel are recommended to work in collaboration toward increasing women’s awareness on NCDs screening during the preconception period. The concerned bodies, especially health extension workers have to mobilize the communities and conduct awareness creation campaigns on NCDs, its risk factors, and the role of preconception care in early identification and management of these problems. In addition, health extension workers are advised to use the existing strategies like women’s developmental armies and women’s conferences to motivate women to improve their lifestyles, nutrition and reduce NCD risk factors.

Health care providers, especially those who are working in the primary health care units are recommended to provide counseling on NCDs and benefits of preconception care for all women of reproductive age groups attending health facilities for different services like family planning, immunization, antenatal care, and medical treatment. In addition, the concerned bodies are advised to create awareness about NCDs and preconception care for men and involve them in maternal and child health care. Indeed, involving important others like religious leaders in maternal and child health care is needed. We also recommend media personnel to give due attention to preconception care and create awareness. Furthermore, researchers, especially in LMICs are advised to conduct studies on preconception care among different women of reproductive age groups (such as women planning to become pregnant, currently married women, etc.) and assess women’s perceptions, beliefs, knowledge, attitudes, intentions, and practices of wellness checkup before they become pregnant.

## Data Availability

The data of the study are available from the corresponding author upon reasonable request.

## Abbreviations

ANC: Antenatal care
CDC: Centers for Disease Control and Prevention
FIGO: International federation of gynecology and obstetrics
NCDs: Non-communicable diseases
SPSS: Statistical package of social science
WHO: World health organization
